# Determinants of central obesity in children and adolescents and associated complications in South Africa: a systematic review

**DOI:** 10.3389/fpubh.2024.1324855

**Published:** 2024-04-23

**Authors:** Cairo Bruce Ntimana, Kagiso Peace Seakamela, Reneilwe Given Mashaba, Eric Maimela

**Affiliations:** DIMAMO Population Health Research Centre, University of Limpopo, Sovenga, South Africa

**Keywords:** central obesity, children and adolescents, socioeconomic status, lifestyle, childhood

## Abstract

**Background:**

Central obesity in children is a global health concern associated with cardiovascular risk factors. In 2019 the World Obesity Federation predicted that in 2025, 206 million children and adolescents aged 5 to 19 will be obese, and the number is estimated to reach 254 million by 2030. There is limited literature on the factors that are associated with the development of central obesity in children. We report a systematic review, aimed to describe the current literature on determinants of central obesity and its associated health outcomes in children and adolescents in the South African population.

**Methods:**

We searched for peer-reviewed studies in Google Scholar, PubMed, and Science Direct search engines, and about seven studies were included. This systematic review has been registered with the International Prospective Register of Systematic Reviews (PROSPERO) (Registration number: CRD42023457012). This systematic review was conducted and reported according to an updated version of the Preferred Reporting Items for Systematic Reviews and Meta-Analyses (PRISMA) guideline. The quality of the included studies was assessed by following guidelines from the Newcastle-Ottawa Scale (NOS). The method considered three main domains: selection, comparability, and outcome across different study designs.

**Results:**

The prevalence of central obesity in children and adolescents by waist-to-height ratio (WHtR) ranged from 2.0 to 41.0%; waist-to-hip [WHR ranged from 10 to 25%; waist circumference (WC) ranged from 9 to 35%]. Central obesity was associated with age, physical inactivity, gender socio, and demographic profiles of the household. Central obesity in children was associated with cardiovascular diseases and mental health issues.

**Conclusion:**

Central obesity in children and adolescents was determined by gender, pubertal development, and age of the parents, households with high socioeconomic status, dietary practices, and overweight/obesity. Given the high prevalence of central obesity in children which can ultimately result in cardiometabolic diseases, cardiovascular risk factors, and mental health issues. This highlights the need for systems, jointly initiated by healthcare providers, policymakers, and the general society aimed at reducing the burden of central obesity such as introducing children and adolescents to health-promoting lifestyles.

## 1 Introduction

Central obesity in children and adolescents has gained public health concern over the years with an increasing prevalence in low-to-middle-income countries (1, 2). The incidence of abdominal obesity is increasing, in both developed and developing leading to high rates of illness and mortality in children and adults (3, 4). In 2019 the World Obesity Federation predicted that in 2025, 206 million children and adolescents aged 5 to 19 will be obese, and the number is estimated to reach 254 million by 2030 (1). Central obesity is linked with the development of non-communicable diseases and mental health issues (5–7). Childhood obesity is reported to likely progress to adulthood increasing the risk of ill health, mortality, and morbidity (8).

Worldwide central obesity is determined by measuring waist circumference which is known to be a better marker for central obesity and a good predictor of cardio-metabolic risks in both adults and adolescents (7, 9–11). Waist circumference (WC) of above 94 cm and 80 cm in males and females, respectively, are indicative of central obesity in adolescents (7, 12). WC percentile ≥95th is also reported as indicative of central obesity (13). A waist circumference to height ratio (WHtR) of 0.5 can be used for early diagnosis of abdominal obesity in children (7, 10, 14, 15). Furthermore, the WHtR cut-off of 0.5 has been validated in multiple countries including South Africa for predicting cardiometabolic risk factors in children and adolescents aged 6–18 years (16). When compared with BMI, WHtR has a stronger association with childhood mortality (10, 17). Central obesity is driven by different factors that include genetics, behavior, and environment (18). The prevalence of childhood obesity is likely to increase due to the increase in urbanization that is pushing more people below the poverty line (19, 20). People with low socioeconomic status are more likely to have poor food choices and food insecurities (21, 22). Reduced protein diet consumption in a population that mostly consumes a carbohydrate-rich diet is linked with visceral fat deposition leading to visceral obesity in children and adolescents (23–25).

Central obesity in children has long-term and short-term outcomes. Short-term, centrally obese children tend to suffer from psychosocial comorbidities such as depression, anxiety, low self-esteem, as well as emotional and behavioral disorders (5–7). Long-term, children with central obesity are likely to have central obesity in adulthood which is linked to the development of cardiovascular diseases, diabetes mellitus, and musculoskeletal disorders, which can lead to disability mortality, and morbidity (5). Treatment of obesity-related comorbidities is difficult, and costly to the health systems. The progression of early childhood overweight/obesity to adulthood increases the risk of cardiovascular risk factors (i.e., atherosclerosis, hypertension, diabetes, and chronic kidney disease) which are on the rise in South Africa (26, 27). The development of single morbidity is linked to the development of secondary morbidity resulting in multimorbidity which is a current global public health concern (28, 29). Therefore, there is a need to reduce the predictors of overweight and obesity at an early age.

To meet the World Health Organization’s (WHO) target of 2025 of “no increase in childhood obesity” it is essential to understand the burden of childhood overweight/obesity in South Africa. Also understanding the burden of abdominal obesity is essential in the South African context, as it may inform resource distribution and policies that sought to reduce the condition by health education, screening, and early intervention. Studies have highlighted the impact of different obesities contributing differently to cardiovascular risk (30–32). Literature has highlighted visceral obesity as a significant predictor of the development of chronic conditions and mental health issues as compared to general obesity (5, 33–35). In this review, we aimed to describe the current literature on determinants of central obesity and its associated health outcomes in children and adolescents in the South African population.

## 2 Materials and methods

### 2.1 Protocol and registration

This systematic review has been registered with the International Prospective Register of Systematic Reviews (PROSPERO) (Registration number: CRD42023457012). This systematic review was conducted and reported according to an updated version of the Preferred Reporting Items for Systematic Reviews and Meta-Analyses (PRISMA) guideline (see Supplementary Table S3) (32).

### 2.2 Search strategy

We systematically conducted a search through Google Scholar, PubMed, and Science Direct databases up to 9th August 2023. The search strategy developed from PubMed was as follows [((((((((((((determinants) OR (effect)) OR (factors)) AND (central obesity)) OR (increased waist circumference)) OR (increased visceral fat)) OR (increased subcutaneous fat)) AND (children)) OR (minors)) OR (adolescent)) OR (teenager)) AND (associated factors)) AND (South Africa)]. The search strategy was adapted for other search engines (Google Scholar, Cochrane, Research4life, and Science Direct). Reference lists of the retrieved studies were also searched to identify additional eligible studies. Gray literature such as dissertations and conference presentations were also searched for relevant studies.

### 2.3 Study selection

To reduce the potential for selection bias, studies were selected based on eligibility criteria by two independent investigators (CBN and RGM). The initial screening procedure comprised a review of the title, abstracts, keywords, and overall objectives of the study, as informed by population, Exposure, Comparator, Outcomes (PECO) guidelines, and eligibility criteria. The investigators individually downloaded studies considered acceptable for review based on screening by title, abstract, and full-text screening. In situations where the two independent investigators did the primary search and study selection, and were not in agreement the third and the fourth investigators (KPS and EM) assessed the studies in question and made a decision.

PECO eligibility criteria.

Population: Children and adolescents.

Exposure: Central/abdominal obesity, visceral obesity.

Comparator: The review included studies that have non-central obesity patients to serve as controls.

Outcomes: Central obesity and cardio-metabolic, non-communication disease, and mental health issues.

#### 2.3.1 Inclusion criteria

The systematic review followed the following inclusion criteria:

- Studies published in South Africa and accessible in South Africa.
- Studies investigating the prevalence and associated risk factors of central obesity in children and adolescents (up to 20 years) conducted from 2010 to 2023;
- Studies that measured central obesity in children using the following methods, ultrasound to measure visceral and subcutaneous fat, waist circumference, waist-hip ratio, and waist-to-height ratio.
- Articles published in English.

#### 2.3.2 Exclusion criteria

The systematic review followed the following exclusion criteria:

- A study conducted in adults;
- Studies that focused on general obesity instead of central obesity;
- Studies not published in South Africa;
- Studies not accessible in South Africa.

### 2.4 Data extraction

The authors (RGM and CBN) independently extracted the following data from eligible studies: name of the first author, year of publication, sample size, age, and determinants of central obesity, associated complications, and interventions. Age was recorded in terms of mean and standard deviation. In cases, median and ranges were given in the study, and this was converted to mean and standard deviation following the guidelines by Wan et al. (36). In cases where a study reported age in different categories, the means and SD were combined using the StatsToDo online tool [CombineMeanSD (statstodo.com)]. The key findings of the studies are summarized in Table 1. Zotero reference manager was used to compile identified studies into a single folder in which the process of duplicate removal/merging was performed. The quality of the included studies was assessed by following guidelines from the Newcastle-Ottawa Scale (NOS). This method considers three main domains: selection, comparability, and outcome across different study designs (44).

**Table 1:** Characteristics of included studies.

Authors and year	Study design	Age (mean and SD) and age range	Race	Residence	Sample size	Prevalence of CO	Definition of central obesity	Determinants	Associated complications	Interventions
Toriola et at. (37)	CS	12.3 ± 1.2 10–16 years	Black	Semi-rural, Limpopo province	1,172 (541 boys and 631 girls)	13.7%	Waist-to-height ratio (≥0.5)	Female gender at age 14–16, teenage years in both sexes, decreased physical activity, sedentary, altered eating patterns, increased fat content in food.	Increased risk of developing cardiovascular and metabolic diseases in childhood and adolescence.	Reduce central obesity in school populations and intensive community-based efforts to prevent central obesity at an early age
Ngwenya and Ramukumba (6)	CS	15.08 ± 1.12 13–19 years	Black	Urban, Gauteng province	175 (110 girls and 65 boys)	5.14%	Waist hip ratio (≥1)	More prevalent in girls and in participants who are overweight	Social effects, emotional well-being, and self-esteem. Liver disease, hypertension, and T2DM, poor quality of life, and poor academic performance.	Interventions aimed to reduce the prevalence of obesity and evaluate the outcomes of such programs. Nutritional interventions.
Monyeki et al. (5)	LS	13.95 ± 1.89 6–14 years	Black	Rural, Limpopo province	1701 (828 girls and 873 boys)	10%	Waist-to-height ratio (≥0.5)	Gender and undernutrition	Cardiovascular and metabolic diseases	Research investigating biological and behavioral risk factors for CVDs and/or diabetes in rural children and adolescents.
Kimani-Murage (38)	CS	15.0 ± 1.47 1–20 years	Black	Rural Mpumalanga province	1848 (945 girls and 945 boys)	15% girls and 2% boys	Waist-height ratio (≥0.5)	Gender, increase in age, post-puberty, high SES households (household highest education), food secure household with high SES (measured using households assets)	NR	Gender-sensitive intervention strategies that take into consideration pubertal development, relative wealth, and related behaviors
Sebati et al. (39)	LS	10.43 ± 2.74 3–16 years	Black	Rural, Limpopo province	25,122 (13,098 boys and 12,024 girls).	3.2% (3–9 years) 0.6% (10–16)	Waist-height ratio (≥0.5)	NR	NR	Early diagnosis and lifestyle education could have a positive impact on the increased prevalence of NCDs. Regular health checkups should be implicated
Kimani-Murage et al. (11)	CS	14.63 ± 3.36 1–20 years	Black	Rural Mpumalanga province	3,489 (1765 girls and 1724 boys)	35%	Waist circumference [≥94 cm for males and ≥80 cm for females, waist-height ratio (≥0.5)]	More prevalent in adolescent girls, increases with sexual maturation and malnutrition	Metabolic diseases	Scaling up of research and program evaluation in order to inform policy on effective intervention strategies that can address the double-prolonged problem of malnutrition
Masocha et al. (40)	LS	14.9 ± 076 14–18 years	Black	Rural North west Province	186 (81 boys and 105 girls)	RN	Waist circumference (≥94 cm for males and ≥80 cm for females)	BMI, elevated blood pressure	Cardio vascular diseases	Programs aimed at increasing physical activity, promoting health dietary practices
Raphadu et al. (41)	LS	4–13 years	Black	Rural Limpopo province	765 (394 males and 371 females)	0.7% boys and 2.0% girls	Waist-height ratio (≥0.5)	Female gender	NR	More studies are needed to focus on the dietary patterns of sodium and potassium and how they can predict abdominal obesity
Debeila et al. (7)	CS	17.93 ± 1.19 1–20 years	Black	Rural, Limpopo province	378 (235 girls and 143 boys)	25% by WHR, 21% by WHtR, 9% by WC	Waist circumference (≥94 for males and ≥80 for females), waist-to-height ratio (≥0.5), and waist-hip ratio (0.90 for males and 0.85 for females)	Girls had higher central or abdominal obesity as compared to boys	Public health implications, metabolic risk factors	Context and culturally appropriate Multi-stakeholder obesity interventions aimed at exposing children and adolescents to a health-promoting environment and nutritional knowledge and improve household vulnerabilities such as a poor socio-economic status
De Lucia et al. (42)	CS	18–19 years	Black	Urban Gauteng province	78 (36 boys and 40 girls)	NR	VAT and SCAT	Girls had higher SCAT and VAT as compared to boys	NR	Future studies should explore the early modifiable determinates of abdominal fat distribution
Kamkuemah et al. (43)	CS	20.73 ± 0.84 15–24 years	NR	Peri-urban, Cape Town Province	87 (66 girls and 21 boys)	41%	Waist-to-height ratio. (≥0.5)	Skipping breakfast, a diet lacking wholegrain, physical inactivity and reported family history of diabetes	NR	Engaging diverse sectors and actors to reduce obesity risk in this priority population group.

CS, cross-sectional studies; LS, longitudinal studies; CVDs, cardiovascular diseases; WHR, waist to hip ratio; WHtR, waist to height ratio; CO, central obesity; NR, not reported; T2DM, type 2 diabetes mellitus; WC, waist circumference.

## 3 Results

**Figure 1:**
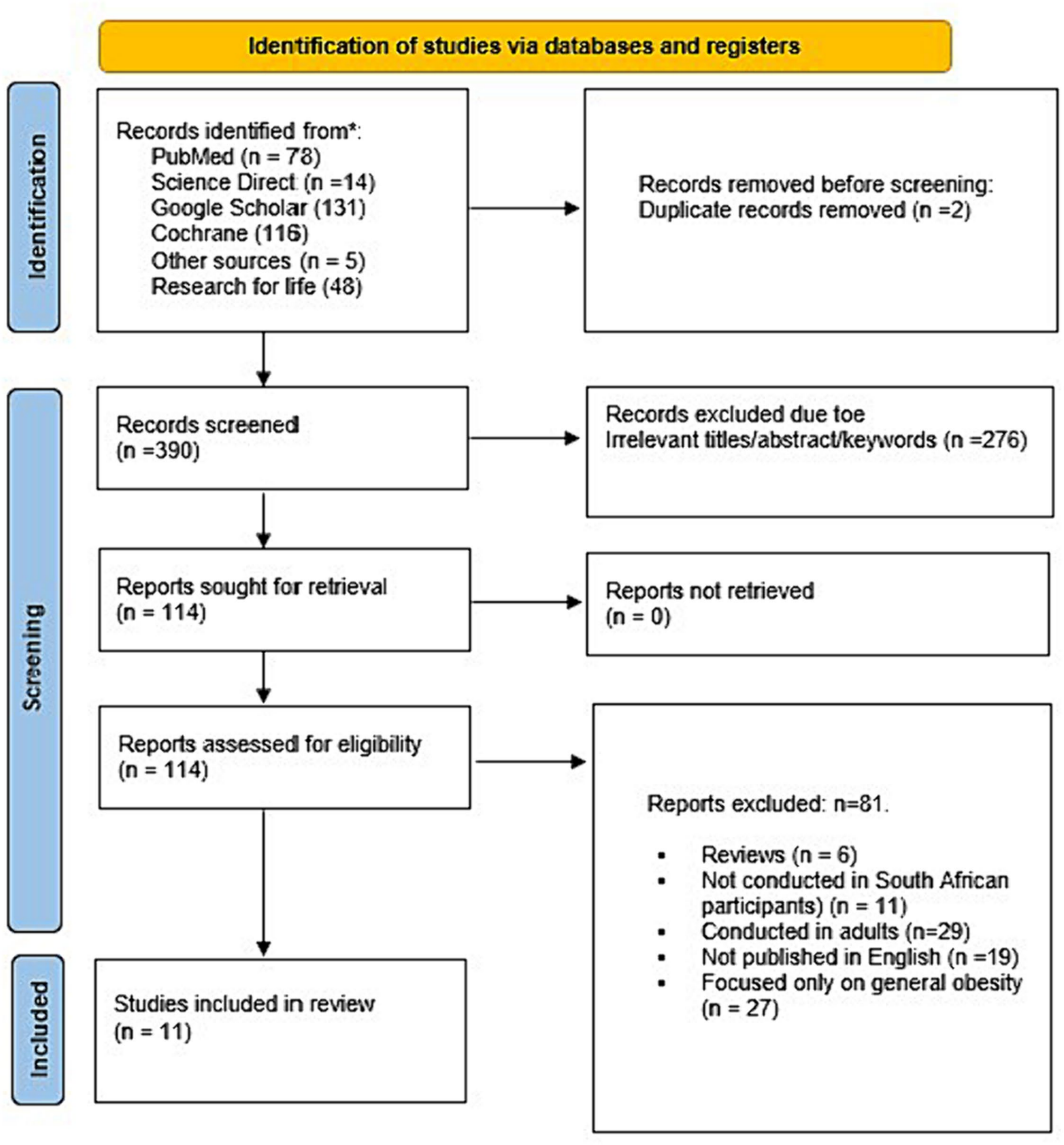
Flow diagram of included studies.

A total of 392 studies were identified by searching databases [Google Scholar (*n* = 131), PubMed (*n* = 78), Science Direct (*n* = 14), Cochrane (*n* = 116), and Research4life (*n* = 48)], and five more studies were found through other sources. About 11 studies met the inclusion criteria for the present study. Other studies were excluded because they were not from South Africa, were conducted among adults and published in languages other than English, or had a singular emphasis on general obesity (Figure 1). Among the included studies, about 7 were cross-sectional studies and 4 were longitudinal studies which were classified as good quality, scoring at least 6 and 7 stars (Supplementary Table S1).

Table 1 presents the general characteristics of included studies sourced from peer-reviewed journals published between 2010 and 2023. Of the included studies, 7/11 (63.6%) were cross-sectional (6, 7, 11, 37, 38, 42, 43) while 4/11 (36.4) were longitudinal studies (5, 39–41). About 5/11(45.5%) of the included studies were conducted in children while 6/11 (54.6%) of the included studies were conducted in adolescents. The age range in the present study was 1–20 years. The present systematic review had a total of 35,001 participants of which 17,921 (51.2%%) were boys and 17,080 (48.8%) were girls. The following methods were used to measure central obesity, waist circumference (7, 11, 38, 40, 43), waist-to-height ratio (5, 7, 37, 43), waist-hip ratio (6, 7, 38, 39, 41), and SCAT and VAT (42). Other included studies used more than one measurement for central obesity namely waist-to-hip ratio, waist-to-height ratio, and waist circumference (5, 7). The waist-to-height ratio (WHtR) showed a prevalence of central obesity ranging from 2.0 to 41.0%; the waist-to-hip (WHR) showed a prevalence of central obesity ranging from 10 to 25%; and the waist circumference (WC) showed a prevalence of central obesity ranging from 9 to 35%.

### 3.1 Determinants of central obesity

#### 3.1.1 Gender and age

Several included studies observed that gender, age, and pubertal/adolescent stages were associated with the development of central obesity with more girls having the highest prevalence of central obesity compared to boys (5–7, 11, 37, 38, 41, 42). In addition, the studies found that boys had the highest proportion of central obesity during the early adolescent stage (age 10 years and above) while the proportion of central obesity in girls increased progressively until reaching its peek between the age of 14–16 years (middle adolescent stage) (5). However, one of the included studies reported that the prevalence of central obesity, measured through the waist-to-height ratio, was lower among girls compared to boys (5).

#### 3.1.2 Physical inactivity

Decreased physical activity (e.g., not active in sports and not participating in athletics) and an increase in sedentarism behavior were also associated with central obesity in children (37, 43). This was noted more in girls than boys (37, 43). The studies reported that boys were generally more active than girls, especially in rural areas (37, 43). One study further highlighted that rural life does not embrace physically demanding tasks anymore thus contributing to low physical activity and sedentarism (43). Cultural norms and values, particularly in rural settings, which prefer healthier bodies among females even during adolescence were also were also reported as a contributing factor for physical inactivity in girls (37, 43).

#### 3.1.3 Dietary practices and general obesity

Diet practices have also been reported as contributing factors to central obesity. These have been reported as altered eating patterns, increased fat content in food, undernutrition, malnutrition, skipping breakfast, and diet lacking wholegrain (5, 11, 37, 43). Children who are already obese/overweight have higher odds of being centrally obese (6).

#### 3.1.4 Parental socioeconomic and sociodemographic status

Children from families with high socioeconomic status (measured using the household’s highest education and household assents), and being in a household that is food secure were also associated with central obesity (45). In addition, children whose parents were aged 50 years and above, with low educational levels were more likely to be overnourished and centrally obese (37, 38, 43).

### 3.2 Complications of central obesity in children

The presence of central obesity in children predisposes them to the early development of liver disease, and cardiovascular and metabolic diseases such as hypertension, and type 2 diabetes mellitus, especially in cases where there is a history of hypertension or diabetes in the family (5–7, 11, 37, 43). Central obesity is also associated with psychological and social effects that impact emotional well-being, and self-esteem leading to poor quality of life and poor academic performance and, public health implications (6, 7).

### 3.3 Central obesity interventions

The included studies identified interventions aimed at combating central obesity in children (5–7, 11, 37, 43, 45). This includes interventions aimed at reducing central obesity in school populations and intensive community-based efforts to prevent central obesity at an early age by engaging diverse sectors and actors to reduce obesity risk in this priority population group (37, 43). Secondly, the studies highlighted a need for gender-sensitive intervention strategies that take into consideration pubertal development, relative wealth, and related behaviors in curbing the rising problem of child and adolescent obesity (45). Context and culturally appropriate obesity prevention interventions. Thirdly, the studies reported that children and adolescents should be exposed to a health-promoting environment to reverse and stop the increased trend of overweight and obesity. Multi-stakeholder interventions focus on improving the nutritional knowledge of children and adolescents to enable them to make healthier food choices and undertake dietary practices like eating breakfast, amidst existing programs. Moreover, modifiable household vulnerabilities such as poor socio-economic status should be considered in the intervention programs. Lastly, Interventions research to reduce the prevalence of obesity and evaluate the outcomes of programs that are in place, nutritional interventions, and research investigating biological and behavioral risk factors for CVDs and/or diabetes in rural children and adolescents.

## 4 Discussion

The prevalence of abdominal obesity in children in South Africa is a significant public health concern. The reviewed literature has highlighted the increasing rates of abdominal obesity ranging from 5.1 to 41.0% in both rural and urban areas (5, 7, 11, 37, 43, 45, 46). The findings of the review are in alignment with other studies conducted in developing countries which reported the prevalence of central obesity in children and adolescents to be rising (3, 4, 47). The reviewed literature reported central obesity in children and adolescents to be determined by gender wherein girls had a higher prevalence of central obesity/abdominal obesity as compared to boys (5–7, 11, 37, 45). The changes in hormonal levels for girls during puberty promote fat deposition in the abdomen, breast, and hips as compared to boys whose hormones promote lean muscle development, the hormonal changes combined with the sedentary lifestyle, and unhealthy food choices that adolescent girls tend to participate in could be the reasons for the findings from the reviewed literature (48, 49). In addition, boys and girls have different energy requirements in accordance with their rates of growth, and they mature at different times.

However, studies in developed countries reported central obesity in children and adolescents to be more prevalent in boys as compared to girls (50). This has been linked to advanced technology. A systematic review and meta-analysis by Ross et al. (51) reported playing video games and spending too much time watching television is associated with central obesity in children (51–53). Dietary patterns have also been identified as a significant risk factor for central obesity. Increased fat content in meals, undernutrition, malnutrition, and a diet lacking in nutritious grains are the dietary patterns, and irregular eating including skipping meals, and an unbalanced diet are associated with central obesity (5, 11, 37, 43). There is a need to enhance the nutritional understanding of parents/guardians, children, and adolescents for them to make healthier food choices and become more involved in healthy dietary practices. These intervention programs should not only be targeted at households with high socio-economic status but also those with low socio-economic status as they are also affected by the poor dietary patterns that contribute to central obesity.

The review reported that children from households that are food secure, with higher levels of education, and more assets were more likely to be centrally obese (45). In agreement with the current study, Misra et al. (54) reported a positive association between high SES (food secured, high education, more assets) of the household with central obesity in children (54). This may be due to the availability of food. There is a need to investigate the influence of relative financial stability and related behaviors (eating and lack of exercise) in addressing the growing problem of central obesity in children and adolescents. Similar has been reported in other developing countries (55). Thus, emphasizing healthier choices and the quality of food and maintaining an active lifestyle.

The reviewed literature reported an increase in age, obesity/overweight, and post-puberty in both genders to be associated with the development of central obesity (45). In agreement with the present study, Chen et al. (56) reported similar findings. Children who are already obese/overweight have higher odds of being centrally obese (6). Similarly, a study by Grigorakis et al. (50) reported obesity/overweight to be significantly associated with central obesity in children (50). In accordance with the review Wagner et al. (57) reported post-puberty to be associated with central obesity in both boys and girls, however, some other studies reported the association to be more common in girls as compared to boys (58, 59). Many potential mechanisms could link adiposity to pubertal timing, but leptin, adipocytokines, and gut peptides are key contributors (57). Genetic diversity and environmental elements like hormone-disrupting substances are other potential mediators (57). To address the growing problem of child and adolescent central obesity, gender-sensitive intervention strategies that account for pubertal development should be explored.

Kimani-Murage et al. (11) indicated that the age of the parent is associated with the development of central obesity in their children. Children living with parents aged 50 years and above have a high likelihood of having central obesity as compared to children who are raised by younger parents (11). There is scarcity of literature about the relationship between parental age and the development of childhood and adolescents’ central obesity. Whereas studies elsewhere have reported that older parents are less likely to engage in daily activities including policing their children’s food choices and physical activities as well as lifestyle of their children which may contribute to obesity. Older parents as compared to younger parents are less informed about health issues which may contribute to their poor dietary patterns in the household (60–62). Given the scarcity of literature on how parental age influences the development of central obesity in children, longitudinal studies are needed to determine the causal relationship between parental age and the development of central obesity in children in South Africa.

The presence of central obesity in children predisposes them to the early development of liver, cardiovascular, and metabolic diseases such as hypertension, and type 2 diabetes mellitus, especially in cases where there is a history of hypertension or diabetes in the family (5–7, 11, 37, 43). Central obesity is also associated with psychological and social effects that impact emotional well-being, and low self-esteem leading to poor quality of life, poor academic performance and, public health implications (6, 7).

In this systematic literature review, two key controversial research topics were identified for the future development of the field. Firstly, literature is scarce on how parental age influences the development of central obesity in their children. Therefore, longitudinal studies should be conducted to determine the causal relationship between parental age and the development of central obesity in children and adolescents in South Africa. Secondly, the role that high SES and technology play in the development of central obesity in children. Future studies should be conducted to determine the role that advancements in technology play in the development of central obesity in children and adolescents and also to investigate the influence of relative financial stability, affluence, and related behaviors (eating and lack of exercise) in addressing the growing problem of central obesity in children and adolescents.

### 4.1 Study limitations and strength

The review included children and adolescents, thus the results cannot be applicable to older individuals. The study’s strength was that we used different databases, and studies were searched independently. Moreover, the overall quality of the included studies was good, as determined by the Newcastle-Ottawa Scale (NOS).

## 5 Conclusion

The review reported the prevalence of central obesity ranges from 2.0 to 41.0% by waist-to-height ratio (WHtR), from 10 to 25% by waist-to-hip ratio (WHR), and from 9 to 35% by waist circumference (WC). Central obesity was determined by gender, households with high SES, age of the parents, dietary practices, and pubertal development. The high prevalence of central obesity in children can ultimately result in cardio-metabolic disease, cardiovascular risk factors, and mental health issues such as memory loss and poor academic performance in school. The review recommends that healthcare providers and policymakers put systems in place that can reduce the burden of central obesity in children for example introducing children and adolescents to a health-promoting lifestyle to bring about change and prevent the increasing incidence of abdominal obesity. Furthermore, healthcare providers and dieticians need to educate parents/guardians, children, and adolescents on healthy dietary patterns.

## Data availability statement

The original contributions presented in the study are included in the article/Supplementary material, further inquiries can be directed to the corresponding author/s.

## Author contributions

CBN: Conceptualization, Methodology, Validation, Visualization, Writing – original draft, Writing – review & editing. KPS: Conceptualization, Methodology, Validation, Visualization, Writing – original draft, Writing – review & editing. RGM: Conceptualization, Methodology, Validation, Visualization, Writing – original draft, Writing – review & editing. EM: Conceptualization, Methodology, Validation, Visualization, Writing – original draft.
